# A multi-omics dataset of heat-shock response in the yeast RNA binding protein Mip6

**DOI:** 10.1038/s41597-020-0412-z

**Published:** 2020-02-27

**Authors:** Carme Nuño-Cabanes, Manuel Ugidos, Sonia Tarazona, Manuel Martín-Expósito, Alberto Ferrer, Susana Rodríguez-Navarro, Ana Conesa

**Affiliations:** 1 Gene Expression and RNA Metabolism Laboratory, Instituto de Biomedicina de Valencia (CSIC). Jaume Roig, 11, E-46010 Valencia, Spain; 2 Gene Expression and RNA Metabolism Laboratory, Centro de Investigación Príncipe Felipe, Eduardo Primo-Yúfera, E-46012 Valencia, Spain; 3 Department of Applied Statistics, Operations Research and Quality, Universitat Politècnica de València (UPV), Valencia, Spain; 4 Microbiology and Cell Science Department, University of Florida, Gainesville, Florida USA; 5 Institute for Food and Agricultural Reserach, University of Florida, Gainesville, Florida USA; 6 Genetics Institute, University of Florida, Gainesville, Florida USA

**Keywords:** Data integration, Metabolomics, Epigenetics

## Abstract

Gene expression is a biological process regulated at different molecular levels, including chromatin accessibility, transcription, and RNA maturation and transport. In addition, these regulatory mechanisms have strong links with cellular metabolism. Here we present a multi-omics dataset that captures different aspects of this multi-layered process in yeast. We obtained RNA-seq, metabolomics, and H4K12ac ChIP-seq data for wild-type and *mip6*Δ strains during a heat-shock time course. Mip6 is an RNA-binding protein that contributes to RNA export during environmental stress and is informative of the contribution of post-transcriptional regulation to control cellular adaptations to environmental changes. The experiment was performed in quadruplicate, and the different omics measurements were obtained from the same biological samples, which facilitates the integration and analysis of data using covariance-based methods. We validate our dataset by showing that ChIP-seq, RNA-seq and metabolomics signals recapitulate existing knowledge about the response of ribosomal genes and the contribution of trehalose metabolism to heat stress. Raw data, processed data and preprocessing scripts are made available.

## Abstract (summary)

**Table:** 

Measurement(s)	RNA • metabolite • histone_modification • epigenetic status
Technology Type(s)	RNA sequencing • nuclear magnetic resonance spectroscopy • ChIP-seq
Factor Type(s)	environmental stress • duration of heat shock • wild type vs Mip6 mutant strain
Sample Characteristic - Organism	Saccharomyces cerevisiae

Machine-accessible metadata file describing the reported data: 10.6084/m9.figshare.11836743

## Background & Summary

Eukaryotic gene expression is a complex process in which the information coded in genes is transformed into functions that support living cells. This process comprises different interconnected steps, which occur in separate compartments and are performed by specific molecular components^1,2^. One of the earlier steps consists of setting up the appropriate epigenetic modifications to allow the expression or repression of specific gene programs^3,4^. These modifications take place mostly on DNA and histones, ensuring access to the proper transcriptional machinery. The specific set of modifications across the genome regulates the final synthesis of the mRNA^5^. These newly synthetized RNA molecules are extensively modified prior to their export to the cytoplasm, where they can be degraded by the mRNA decay machinery, stored in specific foci or translated into proteins^6,7^. Throughout this journey, RNA molecules are guided by RNA-binding proteins that control their fate^7–9^. Finally, the encoded protein products participate in numerous processes, including cellular metabolism where organic compounds are transformed and/or stored. A number of these compounds, such as Acetyl-CoA, glucose or methyl groups, participate, in turn, in chromatin modifications that regulate gene expression.

Our current understanding of transcriptional regulation was largely established using yeast as a model organism. Yeast is an ideal research model for transcriptome research because they exhibit most of the cellular complexity present in eukaryotes and have relatively compact, accessible genomes. However, while the interconnected transcriptional circuit has been studied and accepted by many yeast labs^2,10–15^, these studies usually target aspects of the transcriptional regulation and produce separate results that contribute to the overall transcriptional model. Moreover, different strains, experimental conditions and batches are used by different labs and there are few examples of experimental datasets where all these layers have been measured on exactly the same samples. This poses challenges to the mathematical integration of the data, as methods that rely on the analysis of co-variation patterns will have application restrictions. When the multi-layered data are obtained on the same samples, additional analysis opportunities arise that facilitate the establishment of relationships across regulatory mechanisms.

In this Data Descriptor, we present a yeast multi-omics dataset that features three basic layers of the transcriptional circuit, measured in the same set of samples. These include one epigenetic modification - H4K12ac, a mark for active promoters-, gene expression -RNA-seq- and targeted metabolomics. Moreover, data are obtained for both WT and a *mip6Δ* mutant, in control and heat-shock induced conditions. Mip6 is an RNA-binding protein that participates in RNA export under stress^16^ and consequently is informative of the contribution of post-transcriptional regulation to the adaptation of RNA levels to environmental changes. Taken together, the selection of yeast strains, growth conditions and omics experiments, creates a unique dataset to study the cross-talk between epigenetic, transcriptional and metabolic regulation in response to environmental cues. The availability of multi-omics data on the same set of samples will facilitate the application of powerful statistical approaches that fully leverage paired measurements to propose quantitative regulatory models. A subset of this collection, namely gene expression data for 20 genes that are regulated by stress transcriptional factors Msn2/4, has been published elsewhere^16^.

## Methods

### Experimental design

**Fig. 1:**
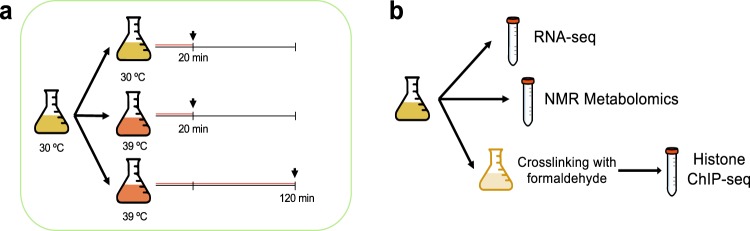
Experimental design and sample management. (**a**) Treatment strategy. For each strain and replicate, a yeast culture flask was grown at 30 °C until the exponential phase, then split into three flasks, each of them receiving a different treatment. (**b**) Sampling strategy. From the same treatment flask, aliquots were collected for RNA-seq, metabolomics and ChIP-seq.

Figure 1 illustrates the experimental desing of our dataset. Panel A describes the strategy to manage sampling and the time-course nature of our experiment. A single, 330 mL culture (either for WT or *mip6*Δ strains) was grown at 30 °C until the exponential phase (OD = 0.7), and this culture was subsequently split across three flasks. One flask was maintained at 30 °C and labeled as time point 0. The other two flasks were incubated at 39 °C for 20 minutes and 120 minutes, respectively, by adding preheated media to rapidly increase the temperature to 39 °C. These last two flasks capture the heat-shock response, while the 30 °C flask serves as a control representing non-stress conditions. The rationale for having two flasks for the 39 °C temperature instead of a single flask sampled at two different time points was to avoid introducing effects related to culture volume. Panel B describes how samples were obtained for omics measuments. Basically, for each of the flasks described above three aliquotes were extracted for RNA-seq, NMR metabolomics, and ChIP-seq analyses. Therefore, the three omics assays were performed on the same cell culture. RNA-seq and NMR aliquotes were obtained first, and the remaining culture was treated to induce cross-linking before collecting the ChIP-seq aliquote. After aliquoting, each tube was inmediatelly frozen and stored at −80 °C.

The process described in Fig. 1 was repeated 4 times to generate four biological replicates. However, due to sample management limitations these 4 replicates were created on two different days. Specifically, biological replicates 1 and 2 were obtained in day 1, and replicates 3 and 4 were obtained in day 2.

### Acquisition and preprocessing of multi-omics data

#### RNA-seq

**Fig. 2:**
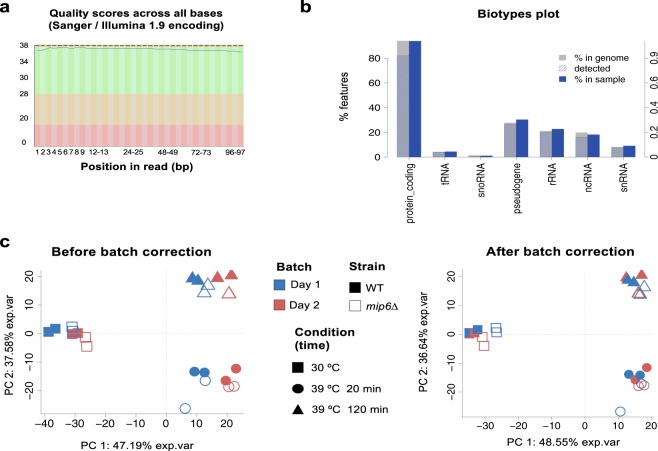
RNA-seq data preprocessing. (**a**) Example of base quality scores across all reads obtained by fastQC analysis, showing uniform read quality. (**b**) Biotype plot of NOISeq package, which indicates that the vast majority of detected features are protein-coding genes. (**c**) Batch effect correction. PCA score plots are represented. The left panel shows raw data where a day-of-culture batch effect is observed. The right plot shows the corrected data where this batch effect has been removed.

Total RNA was isolated by hot acid phenol extraction. RNA integrity was checked with Bioanalyzer (Agilent) and then submitted to a commercial sequencing facility (Macrogen Corea). Sequencing was done with Illumina using the TruSeq protocol. Between 50–60 million reads of 100 bp paired data were obtained from each sample. Raw sequencing data quality was checked by fastQC and good overall quality (Fig. 2a) was observed in all cases. No trimming was deemed necessary. Reads were mapped to the yeast saccer3 genome with Tophat2^17^ and genes were quantified with HTSEQ^18^, *intersection-option*. Supplementary Table S1 shows the number of reads, mapping rate and number of reads in genes for all samples, revealing uniform quantities across the dataset.

The NOISeq^19^ R package was used to perform the quality control of count data. We observed most of reads mapped onto protein-coding genes (>80%), as expected (Fig. 2b). Counts were normalized via TMM^20^ and a low count filtering was applied with the NOISeq *cpm* method (with cpm = 1). Principal Component Analysis (PCA) indicated a slight batch effect for the day of culture growth (Fig. 2c left) that was removed by ARSyN^21^ (Fig. 2c right). In total, we obtained gene expression values for 6,379 genes.

#### Metabolomics

**Fig. 3:**
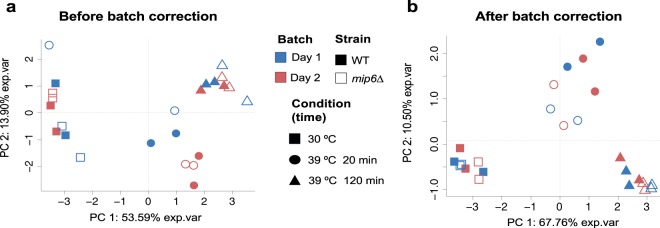
Batch effect correction of metabolomics data. PCA score plots are represented. (**a**) Raw data shows a slight day of culture batch effect for the 20 min 39 °C condition. (**b**) Batch effect corrected data.

Metabolomics measurements were performed on an NMR platform as described in^22^. Basically, metabolites were extracted via chloroform–methanol extraction and the spectra of cell extract samples were recorded on a Bruker AVII-500 using a TCI cryoprobe with spinning at 3,500 Hz. Spectra were processed using Topspin2.16 software (Bruker GmbH, Karlsruhe, Germany). Metabolite identification and assignment were performed with the help of the Human Metabolome Database and 2D NMR experiments. Signal peaks of spectra were normalized considering that the sum of peak areas across all metabolites was constant for every sample, and values for each metabolite were given as a fraction of the total area. A total of 45 compounds were detected, that included 5 sugars, 17 amino-acids, 4 alcohols, 3 vitamin-derivated compounds, 5 carboxylic acids, and other compounds (CMP, NAD, Glutathione, ATP and GMP), plus 3 unidentified metabolites (Table 1). Raw data were log2 transformed and compounds with non-positive measure across all samples were removed, as they were considered to be below reliable detection limit. PCA analysis indicated a small batch effect (Fig. 3a), that was removed by ARSyN (Fig. 3b).

**Table 1:** Metabolites measured by NMR in control and 20′ heat-shock.

		Relative metabolite concentration*			
		WT		*mip6∆*	
Name	Class	30 °C	39 °C 20′	30 °C	39 °C 20′
Acetate	carboxylic acid	66.55	34.73	72.47	63.91
Alanine	amino-acid	30.46	52.78	31.48	51.51
Arginine	amino-acid	20.18	19.45	21.39	19.15
Asparagine	amino-acid	4.37	3.72	4.23	3.67
Aspartic acid	amino-acid	12.00	12.94	12.72	14.04
Glucose	sugar	2.80	2.01	2.55	2.39
Citrate	carboxylic acid	1.32	2.29	1.85	2.67
Ethanol	alcohol	40.55	45.87	45.59	35.47
Fructose 1,6-biphosphate	sugar	2.56	1.08	1.88	1.81
Galactitol	alcohol	6.34	7.29	6.37	5.30
Glutamic acid	amino-acid	69.42	75.83	72.25	68.85
Glutamine	amino-acid	4.90	5.78	5.44	5.11
Glutathione	other	5.02	6.60	4.19	4.15
Glycerol	alcohol	11.10	4.02	10.25	7.20
Glycine	amino-acid	17.21	16.21	20.93	20.01
Glycerophosphocholine	alcohol	27.13	43.10	30.96	37.31
Histidine	amino-acid	3.54	3.84	3.41	3.70
Isoleucine	amino-acid	4.79	4.05	5.59	4.15
Lactate	carboxylic acid	11.21	12.60	12.97	18.25
Leucine	amino-acid	5.73	6.59	5.42	4.61
Lysine	amino-acid	69.70	67.99	70.65	65.10
Myo-inositol	vitamin-derivated	2.24	1.08	1.64	1.79
NAD	other	2.51	1.77	2.45	1.91
Phenylalanine	amino-acid	5.11	5.32	5.39	5.66
Proline	amino-acid	2.57	1.43	1.46	1.68
Serine	amino-acid	2.51	7.54	2.66	5.41
Succinate	carboxylic acid	2.51	1.09	3.17	1.47
Thiamine derivate 1	vitamin-derivated	1.00	1.00	1.01	0.99
Thiamine derivate 2	vitamin-derivated	2.76	2.69	2.59	2.30
Trehalose	sugar	1.11	8.51	1.12	6.15
UDP-glucose	sugar	1.69	1.48	1.96	1.00
Valine	amino-acid	6.69	6.86	7.35	5.90
Beta-D glucoronic acid	carboxylic acid	2.63	2.45	2.77	2.96
Alpha-glucose	sugar	N.D.	N.D.	N.D.	N.D.
ATP	other	N.D.	N.D.	N.D.	N.D.
CMP/CTP	other	N.D.	N.D.	N.D.	N.D.
GMP	other	N.D.	N.D.	N.D.	N.D.
Trypthphane	amino-acid	N.D.	N.D.	N.D.	N.D.
Tyrosine	amino-acid	N.D.	N.D.	N.D.	N.D.
UDP-derivative 1	other	N.D.	N.D.	N.D.	N.D.
UDP-derivative 2	other	N.D.	N.D.	N.D.	N.D.
UDP-derivative 3	other	N.D.	N.D.	N.D.	N.D.

Metabolites measured in this study.

*Average value of four replicates; N..D.: not detected.

#### ChIP-seq

For ChIP-seq samples, chromatin immunoprecipitation was performed as previously described^23^. After cross-linking the cultures for 20 minutes at room temperature with 1% formaldehyde (Sigma), they were quenched with 125 mM glycine for 15 minutes. Subsequently, the cells were collected by centrifugation, split in two aliquots (one for each ChIP) and washed with 25 mL of cold Tris-saline buffer (20 mM Tris-HCl, 150 mM NaCl, pH 7.5) three times. The pellets were frozen in liquid nitrogen and stored at −80 °C until further processing. Cells were disrupted by adding 300 µL of lysis buffer (50 mM HEPES-KOH at pH 7.5, 1 mM EDTA, 140 mM NaCl, 1 mM PMSF and protease inhibitors) and 200 µL of glass beads and vortexing for 13 minutes at 4 °C. The cell extracts were sonicated for 30 minutes in a Bioruptor sonicator (Diagenode) at high intensity using 30 seconds on/30 second off cycles in a 4 °C water bath. The cellular lysate was clarified by centrifugation at 12,000 rpm for 10 minutes at 4 °C and the whole supernatant was used for immunoprecipitation by incubating with magnetic beads (Dynabeads, Invitrogen) bound to anti-histone H4 (Abcam) or anti-histone H4K12ac (Active Motif) antibody for 2 hours at 4 °C. Beads were subsequently washed twice with lysis buffer, twice with lysis buffer supplemented with 360 mM NaCl, twice with wash buffer (10 mM Tris-HCl, pH 8.0, 250 mM LiCl, 125 mM Nadeoxycholol, 1 mM EDTA and 0.5% NP-40), and once with TE buffer. Samples were eluted by adding 50 µL of elution buffer (50 mM Tris-HCl, pH 8, 10 mM EDTA, 1% SDS) to the beads and incubating for 10 min at 65 °C. This step was repeated twice. The samples were incubated overnight at 65 °C to reverse the cross-linking and then incubated with 100 µg/250 µl of proteinase K (Ambion) for 1.5 h at 45 °C. DNA was isolated by phenol extraction. This DNA was sent to Macrogen Corea for sequencing.

**Fig. 4:**
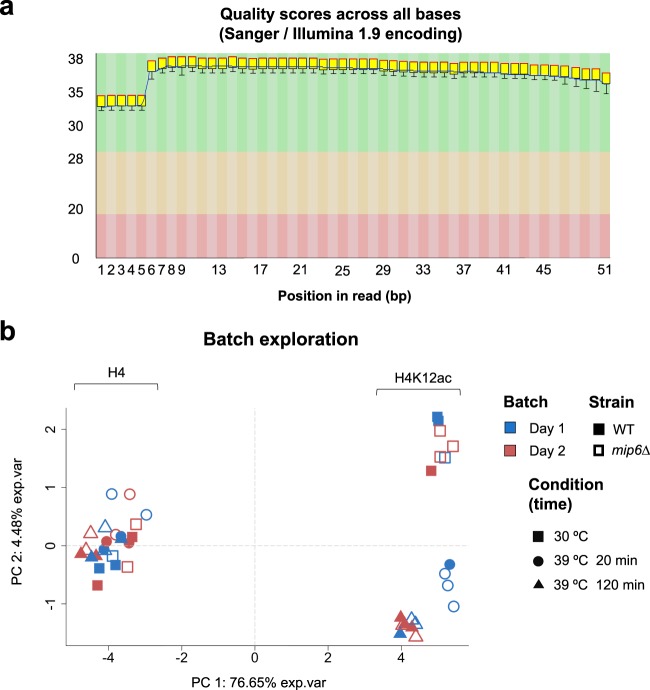
ChIP-seq data preprocessing. (**a**) Example of base quality scores across all reads obtained by fastQC analysis. (**b**) PCA of H4 and H4K12ac data. The first PC indicates the type of ChIP-seq assay, while the second PC reflects the heat treatment in the H4K12ac samples. No batch effect is observed.

Sequencing was done following the Illumina TruSeq protocol. Around 20 million 50 bp reads were obtained for each sample. Note that two ChIP-seq data files were obtained for each sample: H4 and H4K12ac. H4 files contain the reads after purification of total H4 histone and H4K12ac files contain the data associated to acetylation of Lysine 12. Raw sequencing data quality was checked by fastQC and good overall quality (Fig. 4a) was observed in all cases. Trimming of Illumina adapters was performed using Cutadapt^24^. Reads were mapped to the yeast saccer3 genome with Bowtie2^25^. Supplementary Tables S2 and S3 summarize sequencing performance in terms of number of reads and mapping rate for H4 and H4K12ac samples, respectively.

H4 sample of mip6.39.0 was discarded as it showed poor sequencing performance. Macs2 software^26^ was used to call Histone 4 acetylation peaks on the H4K12ac samples alone. Next, a consensus file was generated by merging peaks across all samples using the *merge* command from bedtools software^27^ with default parameters. These consensus regions were used to map back reads of all samples, including H4 samples. Peaks were quantified with HTSEQ^18^, *intersection-option*. NOISeq^19^, R package was used to perform a quality control of count data. Moreover, coverage per base was obtained for both, H4 and H4K12ac samples, using the *genomecov* command from bedtools^26^.

## Data Records

### Raw data

The yeast WT and *mip6*Δ heat-shock multi-omics data have been deposited in different public repositories^28,29^. Table 2 shows a list of the current hosting of raw data files.

**Table 2:** Public repositories hosting the yeast Mip6 data.

Dataset	Database and accession
mRNA-seq	GEO, GSE135568^28^
ChIP-seq	GEO, GSE135568^28^
Metabolomics	MetaboLights, MTBLS1320^29^

Additionally, processed data are also accessible from the Figshare repository, with 10.6084/m9.figshare.c.4716677 ^30^.

## Technical validation

### Validation of dataset replicability

**Fig. 5:**
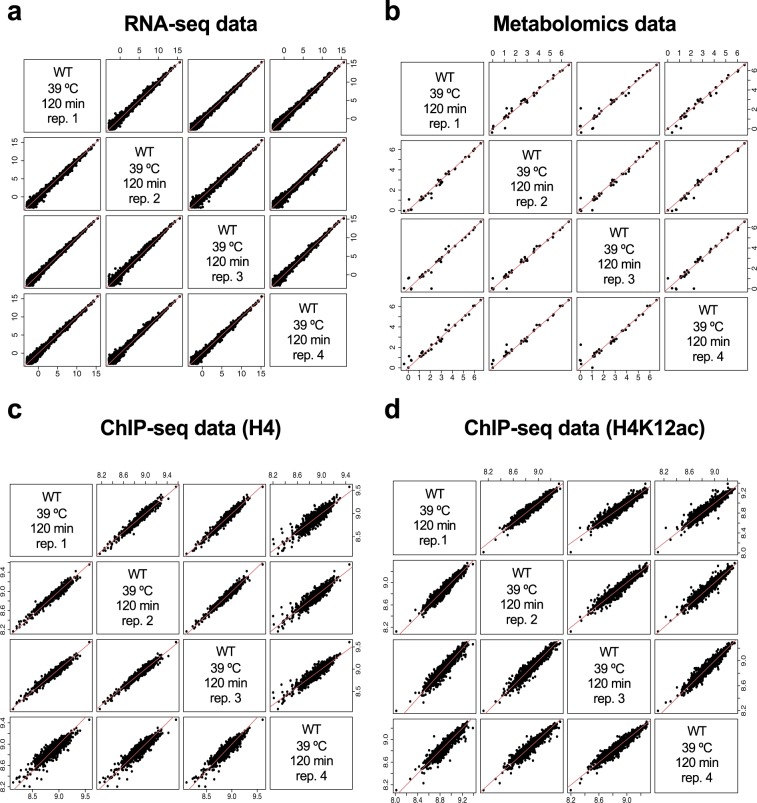
Replicability of processed data. Wild type 39 °C 120 minutes sample is selected as an example. Log2 transformed data are shown. (**a**) RNA-seq. (**b**) Metabolomics. (**c**) ChIP-seq (H4). (**d**) ChIP-seq (H4K12ac). Red diagonal line indicates perfect correlation between samples.

In order to assess replicability, pairwise scatter plots were obtained for RNA-seq data (Fig. 5a), metabolomics data (Fig. 5b) and ChIP-seq data (Fig. 5c,d). Only WT strain replicates are shown as *mip6*Δ strain data behaved similarly. Replicates were highly and equally correlated with each other, and no experimental outliers were detected.

### Validation of biological consistency

Translational repression upon heat-shock occurs in most eukaryotes^31,32^, and it is well known that ribosomal genes rapidly shut down after heat treatment. Moreover, previous studies assessing the impact of heat-shock on yeast cells revealed a protective effect for trehalose^33,34^. We evaluated whether this effect was corroborated by our data.

**Fig. 6:**
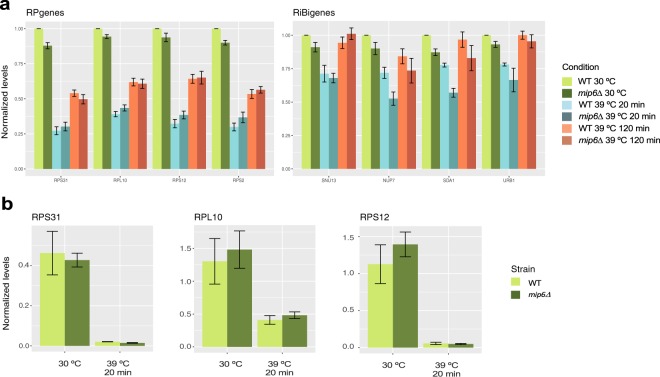
RP and RiBi gene expression levels during heat-shock response. (**a**) Expression levels of RP and RiBi genes (left and right panel, respectively) calculated from RNA-seq at different time-points of the heat-shock. WT and *mip6Δ* samples are depicted in lighter and darker colors, respectively. Each bar represents the mean of four biological replicates with the standard error of the mean (s.e.m.) shown as error bars. All values were normalized to the value of the WT at 30 °C. (**b**) Validation of the RNA-seq experiment results by RT-qPCR from samples incubated under 30 °C and 39 °C for 20 minutes. Each bar represents the mean of three biological replicates with s.e.m. shown as error bars. Values were calculated using the ΔΔCt method.

First, we analyzed ribosomal data. As expected, we found a general down regulation of both ribosomal protein genes (RP genes, Fig. 6a, left panel) and ribosomal biosynthesis genes (RiBi genes, Fig. 6a, right panel) upon a heat treatment at 39 °C. This response was similar for WT and *mip6*Δ strains. The strongest effect was observed after 20 minutes of heat (blue bars), and recovery was observed for all genes after 120 minutes (orange bars). The drastic downregulation of several RP genes after 20 minutes at 39 °C was further validated in independent experiments by q-PCR (Fig. 6b).

**Fig. 7:**
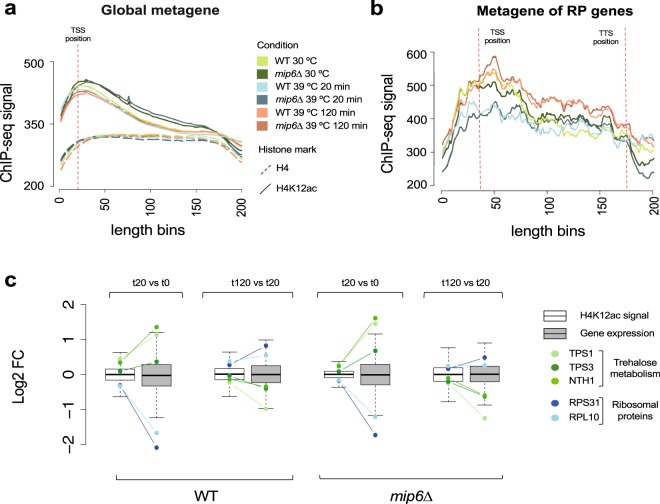
ChIP-seq data correlate with gene expression changes. (**a**) Global metagene indicates that H4K12 acetylation is mostly present at the Transcription Start Site (TSS) of genes. (**b**) Metagene analysis of RP genes reveal acetylation differences across time points. (**c**) Boxplot of log2FC values between consecutive time points for H4 and H4K12ac signals. Positions for marker RP and trehalose metabolism genes are indicated. Although signal distribution for RNA-seq is wider, positions at the data distribution for marker genes are shared between RNA-seq and ChIP-seq data.

It is well stablished that histone modifications modulate gene expression programs^35^. Among other modifications, histone acetylation is considered as a key player in the epigenetic control of gene expression and is associated with transcriptionally active genes^36^. Moreover, a significant deacetylation of H4 was observed after a one hour heat-shock of HeLa cells, being histone H4K12ac affected^37^. To evaluate our ChIP-seq data in relation to heat-shock we first analyzed the composite profile across all genes for the H4K12ac marker (Fig. 7a). In agreement with its role in transcriptional activation, we found a general enrichment of H4K12ac at the Transcription Start Site (TSS) of genes, and, as expected for the heat-shock response, we found consistently lower levels for all 39 °C samples. Moreover, we found a significant reduction of H4K12 acetylation at the TSS of RB genes after 20 minutes of heat-shock (Kolmogorov-Smirnov test *p*.value < 1e-10, Fig. 7b, blue lines), which agrees with the strong down regulation of their expression at this time point (Fig. 6a, blue lines). Notably, H4K12 acetylation levels appeared to be fully restored after 120 minutes (Fig. 7b, orange lines) while gene expression levels were not (Fig. 6a, orange lines). This result suggests that H4K12 acetylation responds more rapidly to heat stress than gene expression.

To determine if this time-dependent heat-response pattern between gene expression and H4K12 acetylation was a general pattern we analyzed the distribution of both types of data. In particular, we obtained the mean read coverage per base at the TSS ± 100  p for the H4K12ac signal in each gene, and computed log2 fold-change values (Log2FC) of the comparison between consecutive time-points. We compared this distribution to the Log2FC of gene expression. This analysis showed that the gene expression response measured by RNA-seq (grey boxes) has a larger dynamic range than H4K12 acetylation measured by ChIP-seq (white boxes), as the distributions are broader in the former (Fig. 7c). Additionally, the transcriptional response at 0′–20′ is overall larger than that at 20’ to 120′. However, the direction of signal change at the gene-level seems to be the same for both omics layers, since the position of selected genes in the Log2FC distributions is similar for RNA-seq and H4K12ac data (Fig. 7c). This was true both for genes that are down-regulated upon heat-shock (RP genes) than for upregulated genes (i.e. trehalose metabolism genes, Fig. 7c). We concluded that a coordinated signal of H4K12ac and gene expression can be inferred from our data, although the magnitude of change in each differs, with RNA-seq data manifesting a larger dynamic range.

**Fig. 8:**
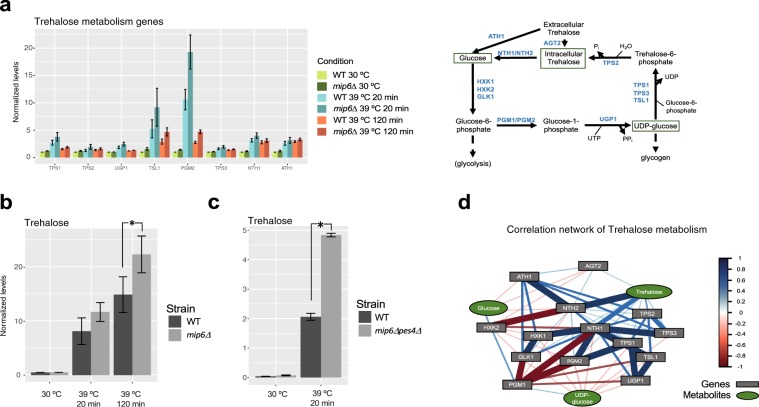
Trehalose metabolism genes and metabolites during heat-shock response. (**a**) Expression levels of trehalose metabolism genes calculated from RNA-seq at different time-points after heat shock. WT and *mip6Δ* samples are depicted in lighter and darker colors, respectively. Each bar represents the mean of four biological replicates with the s.e.m. shown as error bars. All values were normalized to the value of the WT at 30 °C. Lower panel shows a scheme of trehalose metabolism in budding yeast where genes that codify for trehalose metabolism enzymes are represented in blue and metabolites in black. (**b**) Trehalose metabolite levels in WT and *mip6Δ* cells at different time points of the heat-shock. Each bar represents the mean of four biological replicates except for *mip6Δ* 39 °C 20 minutes value, which is calculated from three replicates. Error bars represent the s.e.m. *Indicates one-tailed unpaired Student’s t-test *p*-value < 0.05. (**c**) Trehalose metabolite levels in WT and *mip6Δpes4Δ* cells under 30 °C and 39 °C 20 minutes conditions. Each bar represents the mean of three biological replicates with the s.e.m. shown as error bars. * indicates one-tailed unpaired Student’s t-test *p*-value < 0.05. (**d**) Correlation network of trehalose metabolism. Genes are showed in gray boxes and metabolites are represented using green ellipses. The color intensity and thickness of edges are proportional to the correlation values between nodes. Correlations lower than 0.5 are not showed. For network clarity, some direct associations were omitted if an intermediate node exists to infer such connection.

Figure 7c suggested that expression changes of trehalose metabolism genes in *mip6*Δ cells were larger than in WT. We therefore investigated further this pathway. We confirmed a general -although with different magnitudes- upregulation for genes members of the trehalose metabolism pathway (Fig. 8a). Interestingly, we observe the highest value always for *mip6*Δ cells under these conditions, particularly for *TSL1* and *PGM2* genes, that showed the strongest transcriptional regulation, suggesting that a heat-induced accumulation of trehalose might be larger in the *mip6*Δ versus the WT. We verified this hypothesis by analyzing trehalose levels of our metabolomics dataset (Fig. 8b). We found a strong increase of this metabolite in the treated cells and a significant higher accumulation in the mutant. The metabolomics measurement was further confirmed by an independent analysis, where we measured trehalose levels in a double mutant lacking *MIP6* and its yeast paralogue *PES4*^16^ (Fig. 8c). Finally, network analysis of gene-metabolite levels of the trehalose pathway shows a strong correlation of trehalose with genes using this metabolite either as substrate (*NTH1*, *NTH2*) and product (*TPS2*), suggesting a direct regulation of trehalose levels by these gene products (Fig. 8d).

Taken together, this section shows a biologically consistent and coordinated signal of our RNA-seq, ChIP-seq and metabolomics datasets that agrees with previous findings. Our analysis also suggests a specific role for *mip6* in the metabolic control of the heat-shock response, further supporting the biological interest of the dataset.

## Supplementary information

Supplementary table S1

Supplementary table S2

Supplementary table S3

## Data Availability

Preprocessing scripts for each of the omics datasets are available at the Github repository (https://github.com/ConesaLab/MultiMip6).
